# Adjusting HIV prevalence estimates for non-participation: an application to demographic surveillance

**DOI:** 10.7448/IAS.18.1.19954

**Published:** 2015-11-26

**Authors:** Mark E. McGovern, Giampiero Marra, Rosalba Radice, David Canning, Marie-Louise Newell, Till Bärnighausen

**Affiliations:** 1 Queen's Management School, Queen's University Belfast, Belfast, Northern Ireland, UK; 2 UKCRC Centre of Excellence for Public Health (NI), Belfast, UK;; 3 Wellcome Trust Africa Centre for Health and Population Studies, University of KwaZulu-Natal, KwaZulu-Natal, South Africa; 4 Department of Statistical Science, University College London, London, UK; 5 Department of Economics, Mathematics and Statistics, Birkbeck, University of London, London, UK; 6 Department of Global Health and Population, Harvard T.H. Chan School of Public Health, Boston, MA, USA; 7 Faculty of Medicine, University of Southampton, Southampton, UK

**Keywords:** HIV prevalence, non-participation, missing data, selection bias, Heckman-type selection models, demographic surveillance

## Abstract

**Introduction:**

HIV testing is a cornerstone of efforts to combat the HIV epidemic, and testing conducted as part of surveillance provides invaluable data on the spread of infection and the effectiveness of campaigns to reduce the transmission of HIV. However, participation in HIV testing can be low, and if respondents systematically select not to be tested because they know or suspect they are HIV positive (and fear disclosure), standard approaches to deal with missing data will fail to remove selection bias. We implemented Heckman-type selection models, which can be used to adjust for missing data that are not missing at random, and established the extent of selection bias in a population-based HIV survey in an HIV hyperendemic community in rural South Africa.

**Methods:**

We used data from a population-based HIV survey carried out in 2009 in rural KwaZulu-Natal, South Africa. In this survey, 5565 women (35%) and 2567 men (27%) provided blood for an HIV test. We accounted for missing data using interviewer identity as a selection variable which predicted consent to HIV testing but was unlikely to be independently associated with HIV status. Our approach involved using this selection variable to examine the HIV status of residents who would ordinarily refuse to test, except that they were allocated a persuasive interviewer. Our copula model allows for flexibility when modelling the dependence structure between HIV survey participation and HIV status.

**Results:**

For women, our selection model generated an HIV prevalence estimate of 33% (95% CI 27–40) for all people eligible to consent to HIV testing in the survey. This estimate is higher than the estimate of 24% generated when only information from respondents who participated in testing is used in the analysis, and the estimate of 27% when imputation analysis is used to predict missing data on HIV status. For men, we found an HIV prevalence of 25% (95% CI 15–35) using the selection model, compared to 16% among those who participated in testing, and 18% estimated with imputation. We provide new confidence intervals that correct for the fact that the relationship between testing and HIV status is unknown and requires estimation.

**Conclusions:**

We confirm the feasibility and value of adopting selection models to account for missing data in population-based HIV surveys and surveillance systems. Elements of survey design, such as interviewer identity, present the opportunity to adopt this approach in routine applications. Where non-participation is high, true confidence intervals are much wider than those generated by standard approaches to dealing with missing data suggest.

## Introduction

Accurate HIV prevalence estimates are important for many reasons, including the ability to assess disease trajectories, risk factors and the consequences of infection. Estimates from representative household surveys and demographic surveillance are considered the gold standard for estimating HIV prevalence [1]; however, participation in HIV testing as part of these surveys can be low. There are two main sources of non-participation: respondents may not be tested because they could not be contacted for interview (non-contact) or because they completed the interview but declined consent to test (refusal) [2]. The latter category is typically more common. In the nationally representative Demographic and Health Surveys, recent participation rates in HIV testing range from 67 to 97% [3]. Demographic surveillance sites, which routinely collect longitudinal data on entire communities, have also reported low rates of participation [4–7]. Given that these surveys are an important source of evidence for HIV research and policy, and given the extent of missing data in these surveys, it is important to evaluate the accuracy of existing prevalence estimates and to establish methods to improve accuracy where participation is low. There is evidence that respondents who are HIV positive and know or suspect what their status is are more likely to decline to participate [8–12]. The proportion of these individuals may rise with increasing intensity and frequency of public testing campaigns, which is likely given expanding eligibility for HIV treatment [13], the increasing focus on HIV treatment as prevention, and the recent targets set by UNAIDS for testing, treatment and viral suppression [14].

The use of standard imputation approaches (including single, mean and multiple imputation) [15, 16] or propensity-score reweighting [17] to deal with missing data is only appropriate where the data are assumed to be either missing completely at random (MCAR; absence from the data does not depend on either observed or unobserved characteristics of the respondents) or missing at random (MAR; absence from the data does not depend on unobserved characteristics of the respondents) unless there are appropriate auxiliary variables available. To adjust for missing data in HIV prevalence estimation using imputation, we therefore require the assumption that there is no unobserved variable that is associated with both HIV status and testing. If knowledge of HIV positive status itself affects survey participation, for example because individuals who are HIV positive systematically opt out of testing because they fear disclosure, then HIV status is such an unobserved variable. In addition to the problem of biased point estimates, confidence intervals derived from analysis of cases without missing data or imputation-based models can be too conservative because they fail to acknowledge that the relationship between testing participation and HIV status is uncertain and needs to be estimated.

Heckman-type selection models are an alternative that can be used to correct for selection on unobserved variables [18]. This method can be adopted for estimating HIV prevalence by taking advantage of variation in interviewer quality, which is frequently found in surveys [19]. Good interviewers who obtain higher participation rates are able to persuade some respondents who would normally decline to participate into accepting to test. Under the assumption that interviewer assignment is a function of survey design and independent of respondents’ unobserved characteristics, Heckman selection models will provide estimates of HIV prevalence that correct for selection bias, even if there is some unobserved characteristic of the respondent that is correlated with HIV-positive status and participation. The role of the selection variable (here, interviewer identity) can be viewed as analogous to an instrumental variable.

The goal of this paper is to assess the extent of selection bias in conventional HIV prevalence estimates for the population living in the demographic surveillance area of the Africa Centre for Population Health in rural KwaZulu-Natal, South Africa. Using Heckman-type selection models, we provide new HIV prevalence point estimates and confidence intervals for men and women in 2009 that do not require the MAR assumption to be met.

## Methods

### Setting and data

The Africa Centre carries out a health and demographic surveillance of the entire population of an area in KwaZulu-Natal, South Africa, comprising approximately 90,000 residents in total. Since 2003, a longitudinal population-based HIV surveillance has been nested within the overall surveillance, offering annual HIV testing to all adults aged 15 years or older living in the surveillance area. This predominantly rural location (434 km^2^) also incorporates peri-urban and urban areas. The main language in the area is isiZulu. The district remains one of the poorest in South Africa; in 2006, 77% of households had piped water and toilet access [6]. Over the period 2004 to 2011, HIV prevalence increased substantially, as did local antiretroviral treatment scale-up [7].

Data are collected from households on a semi-annual basis, when a key informant provides information on physical structures, household characteristics and events (including births, deaths, and migration), and individual members and their relationships. For the HIV surveillance, respondents are visited annually by teams of two trained fieldworkers. Written consent is sought for an HIV test; following this step, a blood sample is collected by finger prick, and the dried blood spots are prepared in accordance with UNAIDS and WHO guidelines for HIV testing [6]. For our selection model analysis, we use the anonymized identity code of the interviewer who conducts the interview with the respondent as the selection variable. The blood sample is collected anonymously; only a unique numerical code is retained to link with existing surveillance records. Residents in the surveillance area have good access to rapid HIV testing and results through the public-sector HIV counselling and testing (HCT) infrastructure in this community.

These data have provided information on the evolution of the HIV epidemic and the impact of HIV on the local population for over a decade (see www.africacentre.ac.za, from where the data are publicly accessible). The demographic surveillance sampling procedure, data collection and cohort have been described previously [6, 20].

The analysis in this paper is based on the HIV surveillance conducted during the 2009 calendar year. A total of 37,021 individuals were identified from the Africa Centre database as being eligible for participation in the surveillance. Of these, 7688 were found to have migrated, become sick or disabled or died when consent was sought. A further 2158 residents were found to be ineligible or could not be found, mainly due to incorrect demographic or contact information. Before being asked to take an HIV test, 617 residents declined to participate in the surveillance. In this paper we focus on the 25,392 residents who were successfully contacted to participate in HIV testing. Table 1 demonstrates that 35% of women in this group (5565 respondents) consented to the test, compared to 27% of men (2567 respondents).

**Table 1:** Consent to test for HIV at the 2009 Africa Centre Surveillance cohort by sex

	Women		Men	
		
	*n*	%	*n*	%
Refuse to test	10,242	65	7018	73
Consent to test	5565	35	2567	27
Total	15,807	100	9585	100

The main reason eligible residents did not participate in HIV testing at the Africa Centre is that they declined consent for an HIV test. In 2009, only 5.7% of eligible respondents could not be contacted [5]. The high contact rate is likely a result of the HIV survey operations, which include household revisits at later dates, after working hours, and on weekends. Out-migrants from the Africa Centre community are not considered to be eligible for participation in the HIV surveillance, which is intended to collect data that is representative of the population that currently lives in the community. If the population of interest were redefined to include all people who either live in the Africa Centre community *or* who live outside the community but retain social ties to community members, we would expect true HIV prevalence rates to increase because migration has been found to be a risk factor for HIV [21, 22]. Further data and methodological innovation addressing collection of information from migrants is an important direction for future research.

### Summary of the relationship between interviewer identity and consent to test for HIV

**Figure 1:**
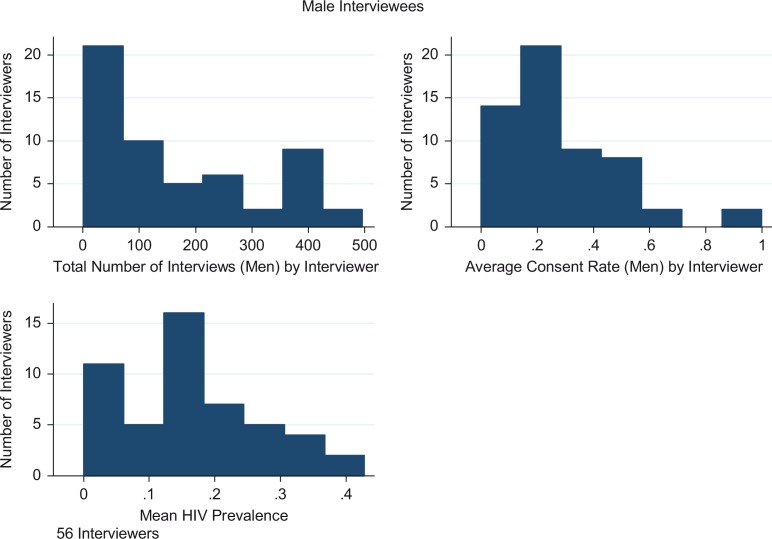
Number of interviews, consent rates and HIV prevalence by interviewer (male respondents).

**Figure 2:**
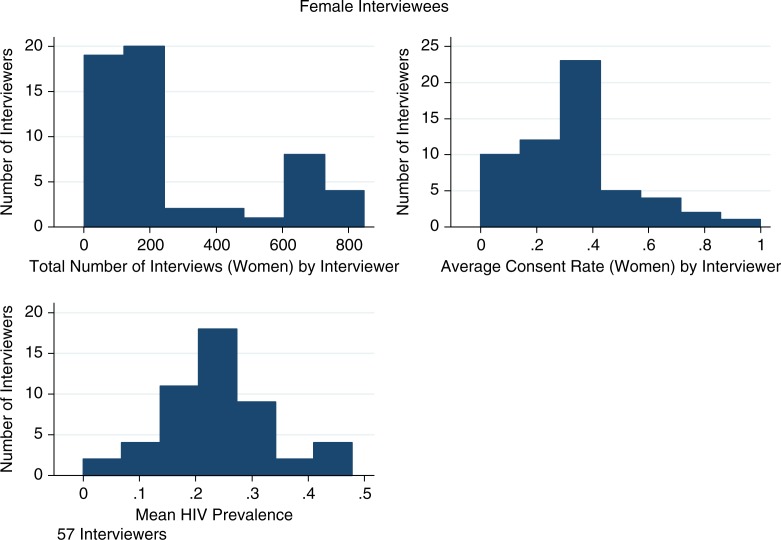
Number of interviews, consent rates and HIV prevalence by interviewer (female respondents).

In the 2009 HIV survey, 57 interviewers asked the 15,807 women who were successfully contacted for consent to an HIV test; 56 interviewers asked the 9585 contacted men for consent to an HIV test. The median number of interviews conducted per interviewer (the number of residents from whom consent to test for HIV was sought by the interviewer) was 174 for women and 127 for men. Median consent per interviewer (the number of residents from whom consent to test for HIV was obtained by the interviewer divided by the number of residents from whom consent to test for HIV was sought by the interviewer) was 25% for men and 33% for women. Good interviewers were equally good at raising consent rates for both men and women. For example, the 25th percentile of interviewer consent is 15% for men and 21% for women, while the 75th percentile for interviewer consent is 39% for men and 40% for women. Among men, HIV prevalence for the median interviewer was 15% (interquartile range [IQR] 10–21%). Among women, the median interviewer found an HIV prevalence of 24% (IQR 18–31%). This information is summarized in Table 2, and histograms of consent rates, number of interviews and HIV prevalence by interviewer are shown in Figures 1 and 2. There is substantial variation in the average prevalence obtained by each interviewer. This variation is exploited in the selection model estimation.

In order to examine the association between having a good interviewer and consent to test for HIV, we ran a logistic regression for consent on an indicator variable for having been interviewed by an interviewer who was over the 75th percentile for consent, adjusting for the other covariates used in the main analysis. We find an odds ratio for consent of having a good interviewer of 2.1 for men (95% CI 2.2–2.8) and 2.1 for women (95% CI 2.0–2.4). Full results of this analysis are shown in Table 3.

**Table 2:** Interviewer statistics for the Africa Centre 2009 HIV survey

	Men	Women
Number of interviewers	56	57
Median number of interviewees per interviewer (25th and 75th percentiles)	127 (32–259)	174 (94–403.5)
Median consent (25th and 75th percentiles)	25% (15–39%)	33% (21–40%)
Median HIV prevalence (25th and 75th percentiles)	15% (10–21%)	24% (18–31%)

Estimates are calculated using one observation per interviewer. For each interviewer, the consent rate is calculated as the number of residents from whom consent to test for HIV was obtained by the interviewer, divided by the number of residents from whom consent to test for HIV was sought by the interviewer. The median HIV prevalence is the median in the distribution of prevalence observed across the participants who consented for each interviewer.

**Table 3:** Predictors of consent to an HIV test

	Women	Men
	
	Logit odds ratio	Logit odds ratio
	
Variables	Consent	Consent
Good interviewer (above 75th consent percentile)	2.17*** (0.09)	2.40*** (0.14)
Interviewer experience (lowest quintile omitted)		
Second quintile	0.96 (0.06)	0.94 (0.08)
Middle quintile	0.94 (0.06)	0.79** (0.08)
Fourth quintile	1.14** (0.07)	0.99 (0.10)
Highest quintile	1.31*** (0.09)	1.35*** (0.14)
Age group (15 to 19 omitted)		
20–24	0.97 (0.09)	0.98 (0.09)
25–29	0.68*** (0.07)	0.79** (0.09)
30–34	0.65*** (0.07)	0.82* (0.10)
35–39	0.65*** (0.07)	0.77** (0.10)
40–44	0.62*** (0.07)	0.80 (0.11)
45–49	0.75*** (0.08)	1.04 (0.15)
50–54	0.83* (0.09)	1.17 (0.17)
55–59	0.87 (0.10)	1.34* (0.21)
60 +	0.92 (0.10)	2.03*** (0.28)
Type of location of residence (*urban* omitted)		
Peri-urban	1.07 (0.07)	1.12 (0.11)
Rural	2.18 (2.56)	0.36 (0.29)
Distance to nearest clinic (≤1 km omitted), km		
1–2	0.94 (0.07)	0.80** (0.08)
2–3	0.92 (0.08)	0.77** (0.09)
3–4	1.02 (0.09)	1.01 (0.13)
4–5	1.18 (0.12)	1.12 (0.16)
5+	1.37*** (0.16)	1.62*** (0.26)
Distance to nearest secondary school, km		
1–2	0.99 (0.05)	0.99 (0.07)
2–3	1.09 (0.07)	1.08 (0.10)
3–4	0.97 (0.08)	0.98 (0.12)
4–5	0.96 (0.11)	0.80 (0.15)
5+	0.65** (0.12)	0.72 (0.20)
Distance to nearest primary school, km		
1–2	1.22*** (0.05)	1.17** (0.07)
2–3	1.16** (0.08)	1.18 (0.12)
3–4	1.25 (0.21)	0.91 (0.25)
Distance to nearest Level 1 road, km		
1–2	0.97 (0.07)	1.03 (0.10)
2–3	0.84 (0.10)	1.11 (0.18)
3–4	0.87 (0.13)	1.04 (0.22)
4–5	0.75* (0.12)	0.95 (0.20)
5+	0.55*** (0.09)	0.70* (0.15)
Distance to nearest Level 2 road, km		
1–2	0.91** (0.04)	0.88* (0.06)
2–3	0.96 (0.06)	0.81** (0.08)
3–4	1.05 (0.10)	1.03 (0.13)
4–5	1.44*** (0.19)	1.58** (0.29)
5+	1.35 (0.26)	3.11*** (0.96)
Marital status (*married* omitted)		
Polygamous	1.10 (0.13)	0.69* (0.15)
Divorced/separated/ widowed	0.95 (0.06)	1.23 (0.22)
Engaged	1.34*** (0.15)	1.00 (0.17)
Never married	1.04 (0.06)	1.71*** (0.16)
Under legal age	0.90 (0.10)	1.91*** (0.25)
Missing/other	0.67 (0.34)	0.41* (0.19)
Mother alive (*dead* omitted)		
Alive	1.01 (0.05)	0.94 (0.08)
Missing/other	0.43* (0.19)	1.26 (0.48)
Father alive (*dead* omitted)		
Alive	1.00 (0.06)	0.90 (0.07)
Missing/other	0.91 (0.22)	0.78 (0.24)
Have electricity in house (*yes* omitted)		
No	0.91 (0.06)	0.95 (0.09)
N/A	1.02 (0.23)	1.08 (0.34)
Missing/other	1.35 (0.75)	0.46 (0.25)
Type of fuel in house (*electric* omitted)		
Coal/wood	1.04 (0.06)	0.82** (0.07)
Gas	1.03 (0.09)	0.87 (0.11)
Other	1.06 (0.13)	0.80 (0.13)
Missing/other	0.92 (0.21)	0.43** (0.14)
N/A	0.75 (0.42)	1.09 (0.60)
Household asset quintile (lowest omitted)		
Second	0.89* (0.06)	1.12 (0.10)
Third	0.88* (0.07)	0.98 (0.10)
Fourth	0.79*** (0.07)	0.83 (0.10)
Fifth	0.71*** (0.06)	0.73*** (0.09)
Missing/other	0.94 (0.19)	1.36 (0.36)
Education (*none* omitted)		
Primary	1.09 (0.07)	0.90 (0.10)
Junior secondary	0.95 (0.07)	0.88 (0.10)
Upper secondary	0.71*** (0.05)	0.70*** (0.07)
Unknown	0.77*** (0.06)	0.75** (0.09)
Missing/other	0.55*** (0.08)	0.86 (0.16)
Running water in house	1.09* (0.06)	1.08 (0.08)
Inside toilet	0.83* (0.09)	1.10 (0.15)
Constant	1.09 (0.26)	0.39*** (0.13)
Observations	15,807	9585

Robust standard errors in parentheses;

*** *p*<0.01

** *p*<0.05

* *p*<0.1; coefficients shown are odds ratios from a logistic regression model for consent to test. In addition to the variables shown in the table, the models also control for location of residence (Isigodi) fixed effects and month of interview, which are not shown for reasons of space. Column 1 is for women only; Column 2 is for men only. The “good interviewer” variable is defined as having been interviewed by an interviewer who obtained an overall consent rate above the 75th consent percentile. For each respondent in the sample, the interviewer consent rate is calculated as the consent rate among that interviewer's other respondents, excluding whether that respondent consented or not (in order to avoid a mechanical correlation between own consent and interviewer-level consent). Interviewer experience is calculated as the number of interviews conducted in the 2009 surveillance by a respondent's interviewer prior to the respondent's own interview.

To further increase our understanding of interviewer performance in eliciting consent to HIV testing, we examined the relationship between interviewer experience and consent rates within the survey itself. We determined how many interviews an interviewer conducted in the 2009 HIV surveillance before contacting a particular survey respondent. We find that interviewers with a greater number of previous interviews were more likely to obtain consent in this next interview. Among the sample of female respondents, the median number of prior interviews conducted by their interviewer was 196, and among the sample of male respondents the median number of prior interviews conducted was 128. The relationship between interviewer experience and consent appears to be non-linear. For female respondents, consent was 36% for interviewers in the bottom quintile of experience, 33% in the middle quintile and 37% in the top quintile. For male respondents, consent was 31% in the bottom quintile, 23% in the middle quintile and 29% in the top quintile. To explore this issue further, we included interviewer experience quintile as a predictor of consent in the analysis shown in Table 3. We find that having an interviewer in the highest experience quintile raises the probability of a respondent consenting to test by 31% for women and 35% for men. Including interviewer experience did not affect our estimates of the association between interviewer consent percentile and the individual's propensity to consent to test. Further research is needed to explore the mechanisms underlying the relationship between interviewer experience and consent. For example, these results could reflect a form of learning by doing or the recruitment and retention process implemented by the survey manager.

### Selection model methodology

Heckman-type selection models estimate the selection process and the outcome simultaneously. By directly estimating the correlation between participation and the outcome, under two standard assumptions this method has been previously used to account for missing data which violate the MAR assumption [3, 23–26]. The approach involves modelling consent to test for HIV using a set of observed characteristics (such as age, marital status and household characteristics), modelling HIV status using a set of observed characteristics, and estimating both equations simultaneously in a bivariate probit framework using maximum likelihood. The first assumption, which has previously been required for Heckman-type selection models to provide asympotically unbiased estimates of HIV prevalence, is that the error terms in both the consent to test and HIV status equations are distributed as bivariate normal. This is a strong assumption which has been criticized as being arbitrary, and is a serious limitation of previous implementations of this approach because it cannot easily be tested. We do not observe the true distribution of the error terms, and misspecification of this distribution could result in bias [27, 28]. In this paper we use a copula approach where we allow the error terms to be derived from a variety of different parametric distributions, and therefore our results do not depend on this assumption [29].

The second assumption required for Heckman-type selection models is that there is a selection variable that predicts consent to test but not HIV status. In this case, we use interviewer identity, as interviewer identity is highly correlated with whether the respondent consents to test for HIV. Moreover, as interviewer assignment is mainly a feature of survey design rather than the characteristics of the respondent, it is unlikely that the interviewer a respondent is assigned to is associated with whether the respondent was HIV positive or not. Interviewer identity is, therefore, used as a predictor of consent to test for HIV in our model, but not as a predictor of HIV status.

The issue of selection bias arises because we only observe HIV status if individuals consent to test. Therefore, our approach explicitly considers consent and HIV status simultaneously via estimation of a selection equation (whose outcome is consent to test for HIV) and a substantive equation (whose outcome is HIV status) [30]. Following Dubin and Rivers [31] and Bärnighausen *et al*. [23], we predict both HIV surveillance participation and HIV status by combining the available data from the household, individual and HIV questionnaires, such that the dummy variable indicator for consent for respondent *i* with interviewer *j* (Consent_*ij*_, which is modelled as a function of a latent consent variable $\text{Consen}{\text{t}}_{ij}^{*}$, reflecting propensity to test) is given by the following equations:

1 Consentij*=Xij'β+Zj'α+uij,i=1…,n; j=1…,j

2 $$\text{Consen}{\text{t}}_{ij}=1\text{ if Consen}{\text{t}}_{\text{ij}}^{*}>0,\text{Consen}{\text{t}}_{ij}=0\text{ otherwise}$$

where *X*_*ij*_ is a vector of control variables and *Z*_*j*_ represents the interviewer effects. The control variables include the following: age group, location of residence (Isigodi), type of location of residence (urban/rural/peri-urban), distance to nearest clinic, distance to nearest secondary school, distance to nearest primary school, distance to nearest Level 1 road, distance to nearest Level 2 road, marital status, education, mother/father is alive, electricity in home, fuel in home, toilet in home, water in home, household asset index and month of interview. The relationship between these variables and consent is shown in Table 3. Similarly, HIV status (HIV_*ij*_, also modelled as a function of a latent variable, $HIV_{ij}^{*}$) is given by the following equations:

3 HIVij*=Xij'γ+εij

4 $$\text{HI}{\text{V}}_{ij}=1\text{ if HI}{\text{V}}_{ij}^{*}>0,\text{ HI}{\text{V}}_{\text{ij}}=\text{0 otherwise}$$

5 $$\text{HI}{\text{V}}_{\text{ij}}\text{ observed only if Consen}{\text{t}}_{\text{ij}}=1,\text{missing otherwise}$$

The same independent variables used in Equation 1 are present in Equation 3, apart from the fixed effects for interviewer identity, which is the key selection variable that only predicts consent [18]. The bivariate probit model jointly estimates the two equations by maximum likelihood. In the standard Heckman-type selection model, the error terms in both equations (*u*_*ij*_,*ɛ*_*ij*_) are distributed as bivariate normal. Therefore, the main parameter of interest in the estimation of HIV prevalence is *ρ*, the correlation between testing and HIV status (*ρ* = corr(*u*_*ij*_,*ɛ*_*ij*_)). In our approach, we relax this assumption by allowing for a variety of different dependence structures using copula functions [29]. Table 4 gives results from the copula model that has the best fit [as measured by the Akaike information criterion (AIC)]; however, our estimates are similar regardless of how the dependence structure is specified. Provided the assumptions outlined above are met, these selection model prevalence estimates will be asymptotically unbiased even when respondents chose not to participate in testing on the basis of unobserved characteristics that are associated with HIV status, or on the basis of HIV status itself [32]. This feature of the results generated by selection models is in contrast to results obtained using analysis based only on those individuals with a valid HIV test, or imputation methods, which assume that missing data are MAR [15, 33].

**Table 4:** Estimates of HIV prevalence

Model	HIV prevalence	95% CI	
Men			
Cases with valid HIV test	16	15	17
Imputation	18	16	21
Heckman selection model (interviewer)	25	15	35
Women			
Cases with valid HIV test	24	23	26
Imputation	27	26	28
Heckman selection model (interviewer)	33	27	40

CI, confidence interval. The following variables are included as predictors of consent to test for HIV and HIV status: age group, location of residence (Isigodi), type of location of residence (urban/rural/peri-urban), distance to nearest clinic, distance to nearest secondary school, distance to nearest primary school, distance to nearest Level 1 road, distance to nearest Level 2 road, marital status, education, mother/father is alive, electricity in home, fuel in home, toilet in home, water in home and household asset index. The first row is the mean prevalence among the sample who consent to test and have a valid HIV test (complete case analysis). The second row imputes HIV prevalence for those who refused consent using the covariates described above. Row 3 implements a Heckman selection model for HIV status and consent to an HIV test using interviewer fixed effects. We show results from the copula selection model with the best fit as measured by the AIC, which for both men and women is the Gaussian copula (equivalent to assuming the error terms are drawn from the bivariate normal distribution). The confidence interval for the imputation model is based on five imputations. The confidence interval for the Heckman selection model is based on the delta method.

### Role of the copula in modelling dependence structure

The use of copulae to model the relationship between an outcome of interest and survey participation allows for a more flexible way of describing dependence and relaxes a key assumption of the original selection model. Finding that one particular copula is the best fit does not in principle depend on whether selection bias is present in the data. For example, in theory it is possible to find the same magnitude of selection bias using any copula, because all copulae allow for unmeasured dependence. A finding that a symmetric copula (such as the Gaussian and Frank copulae) is the best fit could result in an upward adjustment to HIV prevalence, a downward adjustment, or no adjustment, and the same holds for asymmetric Archimedean copulae (including the Joe, Gumbel and Clayton copulae), depending on the degree of rotation.

The use of copulae in selection models is important for two reasons. First, if the underlying structure of the dependence in the data is not Gaussian, then imposing the Gaussian copula (which is equivalent to the standard selection model, which assumes bivariate normality) can result in biased and inefficient estimates of HIV prevalence [27]. The introduction of alternative copulae allows us to assess whether other dependence structures affect results from the model. Second, the copula approach is more likely to accurately reflect the underlying behavioural mechanism of interest. The Gaussian copula imposes the assumption that the dependence structure is symmetrical. In the context of the HIV example and the case of negative selection bias, this structural assumption implies that those who are the most likely to be HIV positive are those who are the least likely to test *and* that those who are least likely to be HIV positive are the most likely to test (and vice versa for positive selection bias). However, it is possible that selection bias is concentrated among those who are most likely to be HIV positive – perhaps because they have the greatest incentive to decline to test – while there is no association between HIV status and testing behaviour for those who are likely to be HIV negative. In this case, dependence would be concentrated in one tail of the distributions for HIV status and testing participation – a behavioural mechanism that the standard selection model assuming bivariate normality would fail to reflect accurately.

The copula approach is very flexible and can incorporate both positive and negative selection bias, which can be symmetrical or asymmetrical. In the HIV case, we expect negative dependence because those who are HIV positive can be expected to be less likely to test; however, there may be exceptions to this rule and in other contexts we could expect positive selection bias. Therefore, when implementing the copula approach for missing data, a practical recommendation for researchers is to first use a model with a symmetric copula such as the Gaussian. Then, if negative selection bias is found, additional asymmetric copulae allowing for negative dependence can be fit (e.g. the 90° and 270° rotated Joe, Clayton and Gumbel copulae). The preferred model will be the copula with the lowest AIC. Alternatively, if positive selection bias is found, additional asymmetric copulae that allow for positive dependence can be fit (e.g. the 0° and 180° rotated Joe, Clayton and Gumbel copulae). Again, the preferred model will be the copula with the lowest AIC.

In what follows, we compare point estimates and confidence intervals for HIV prevalence from a number of different approaches. First, we calculate HIV prevalence using complete cases (those who consent to test for HIV), ignoring the missing data. Second, we implement an imputation model where we predict HIV status for those who decline to consent to test based on their observed covariates. Finally, we use our copula Heckman selection model based on interviewer effects, which accounts for selection on unobserved characteristics.

## Results

Our main results for HIV prevalence are presented in Table 4. The male HIV-prevalence point estimate from the imputation-based model of 18% is comparable to the complete case analysis (only those who consented to test, ignoring the missing data) estimate of 16%. The confidence intervals for these conventional estimates are two to five percentage points wide and assume that the correlation between testing and HIV status is zero (conditional on observed characteristics). In contrast, the point estimate for the selection model is 25%, which is nine percentage points higher than the complete case estimate. However, the confidence interval is much wider (20 percentage points), and thus for men we cannot reject the null hypothesis that the selection model HIV prevalence is the same as the complete case prevalence (16%). Therefore, despite suggestive evidence from the point estimate, from a statistical point of view we cannot reject the null hypothesis that there is no selection bias.

For women, the complete case analysis suggests a population prevalence of 24%, while the imputation-based analysis suggests a prevalence of 27%. However, the selection model estimate is 33%, also nine percentage points higher. As with men, the selection model confidence interval is much wider (23 percentage points) than conventional confidence intervals. However, for women we can reject the null hypothesis that the selection model HIV prevalence is the same as that for the complete case analysis, which provides evidence of selection bias.

## Discussion

Participation rates in HIV testing can be low, and there is evidence that some individuals select not to participate on the basis of factors associated with HIV status [8–12]. In this case, standard imputation models are unlikely to generate unbiased HIV prevalence estimates [33]. Studies that implement Heckman selection models, which are robust to missing data that are not MAR, have confirmed that these point estimates can be affected by selection bias [3, 23, 25, 26, 34]. We applied an interviewer selection model procedure to data from the 2009 Africa Centre HIV surveillance and found moderate selection bias for women, but less clear evidence for men. Just as importantly, our new confidence intervals, which corrected for uncertainty in estimating the relationship between testing and HIV status, were much larger than those based on the usual analytic standard errors.

There are two main implications of these large confidence intervals. First, the signal of the data is limited when either consent or contact rates are low because it is more difficult to precisely estimate HIV prevalence. Second, it is therefore critical to ensure high overall participation rates in HIV surveys. In the Africa Centre in 2009, the overall response rate was the lowest recorded in the history of this surveillance, and since then a number of approaches aimed at raising consent rates have been evaluated, including offers of anonymized pooled testing and a gift intervention [35, 36]. The gift intervention substantially raised consent rates in the surveillance population, and since 2015 has been adopted as part of the routine surveillance approach.

We compared our results to other estimates of HIV prevalence in the province of KwaZulu-Natal, where this study took place. The antenatal care HIV prevalence estimate for women was 40% in 2010 [37], while a national HIV survey found an overall prevalence of 17% in 2012 [38]. Cohort studies also show high rates of infection in KwaZulu-Natal around this time period. A prospective study conducted from 2004 to 2007 found that, among volunteers aged 14 to 30, HIV prevalence was 36% in women recruited from a rural clinic, and 59% in women recruited from an urban clinic [39]. Among sexually active women screened for enrolment into three HIV-prevention studies between 2002 and 2005, HIV prevalence was found to be 43% [40]. In a recent population-based survey, overall prevalence in two districts was estimated to be 25% among those aged 15 to 59 [41]. At another health and demographic surveillance site in KwaZulu-Natal, Agincourt [42], HIV prevalence in 2010 to 2011 for all those over the age of 15 was found to be 19% (11% for men and 24% for women) [43]. In an analysis using a selection model approach, some evidence of selection bias was found at the Agincourt site [24], although the correction was lower in magnitude than the correction estimated in this paper. Using data from the Africa Centre, HIV prevalence among community residents aged 15 to 49 was found to have risen from 21% in 2004 to 29% in 2011 [7]. These authors used multiple imputation to assess the sensitivity of results to the treatment of missing data. In addition, using a procedure where the HIV status of those who refused in any given year was replaced with their HIV status if they participated in testing within a three-year window, they found that HIV prevalence estimates were essentially unchanged. In our estimates in this paper, while there is some indication of selection bias for women, the size of the correction is relatively modest. Therefore, this analysis shows that HIV prevalence point estimates based on conventional approaches using the Africa Centre data are quite accurate. Nevertheless, it is important to conduct further research to establish whether this finding holds over time and across sub-groups of respondents.

When comparing differences in response rates across surveys, it is important to consider all forms of missing data. In the context of HIV surveys, missing information on HIV status can arise from not being able to contact eligible households to request their participation, eligible households that are contacted declining to participate, eligible residents of the consenting households not being found for contact and, finally, eligible residents of consenting households declining to test for HIV. In the case of the Africa Centre, virtually all eligible households were contacted and agreed to participate in the HIV surveillance. Moreover, almost all eligible residents were successfully contacted to request their participation in testing (e.g., 94.3% in 2009 [5]). Therefore, practically all missing data at the Africa Centre arise through individuals directly refusing to test. In some cases at least, failure to contact the individual may be an implicit form of non-consent by that person, and it is likely that if those individuals who were not contacted were actually found and asked to test for HIV they would have had higher rates of non-consent.

While individual-level consent rates are higher in some HIV surveys than those in the Africa Centre in 2009 [38, 41, 43], when all forms of missing data are incorporated into an overall response rate for those who participated in testing, most HIV surveys in South Africa tend to find a high level of missingness. Therefore, there is large potential for selection effects to bias HIV prevalence estimates in South Africa and other countries where overall response rates are low. For example, 66% of eligible residents were contacted as part of the Agincourt health and demographic surveillance system in 2010 to 2011 [43]. Of the 66% who were successfully contacted, 87% agreed to participate in HIV testing, which gives an overall non-missing response rate of 66%*87%=57%. In a national HIV survey conducted in South Africa in 2012, 85% of eligible households participated in the survey (15% either refusing or not being successfully contacted) and 68% of eligible residents in these households participated in HIV testing (32% either refusing or not being successfully contacted) [38]. Conservatively assuming one eligible individual per household, this gives an overall non-missing response rate of 85%*68%=58%. Given that the overall extent of missing data is high in HIV surveys in South Africa, but that the reason for missingness varies across sites, it is important for future research to establish explanations and mechanisms for these differences, especially in view of the recent UNAIDS target of increasing testing rates to 90% by 2020 [14].

Our estimates indicate potential sex differences in the mechanisms leading to survey participation, which is consistent with previous findings from sub-Saharan Africa [3]. However, it is difficult to be definitive about this result in our data because the extent of selection bias appears similar in men, but it is measured with greater uncertainty. This finding may reflect that for women in South Africa, disclosure of HIV status is potentially more damaging for groups with less social power, and women may be less socially powerful than men in this type of community [44]. There are several reasons why this social differential would be expected to result in less precise estimates of selection bias among men than among women. Disclosure of HIV-positive status (either voluntarily or involuntarily) to partners is likely more harmful for women than for men [45, 46], and surveillance participants may not fully accept the confidentiality of the HIV test given [47]. In contrast, the consequences of partner disclosure for men are likely to be less impactful [44, 48]. Therefore, in women consent may be more likely to be driven by HIV status and greater fear of disclosure, while for men HIV status may not be as significant a driver of HIV testing consent, making it more difficult to statistically detect selection bias among men.

Even though good interviewers appear to be similarly persuasive for men and women, we find less evidence that the men who are persuaded to test by good interviewers are more likely to be HIV positive. This finding is not inconsistent with our approach: there is no necessary relationship between the persuasiveness of good interviewers and the change in HIV prevalence estimates based on Heckman selection models. The association between interviewer identity and consent to test needs to exist for Heckman selection models to be able to identify and control for selection bias. However, if selection bias is absent, this approach will not lead to any correction in overall HIV-prevalence estimates, because prevalence estimates for those who do not consent will not differ from those who do consent.

### Limitations

This study has a number of limitations. First, our finding that interviewer identity is highly correlated with consent has implications for surveillance operations, as it implies that raising the ability of less effective interviewers could substantially increase HIV-testing participation rates. Unfortunately, we only had access to an anonymized identity code representing interviewer identity and did not have data on interviewer characteristics (such as sex and age). Establishing why some interviewers are more persuasive than others is an important direction for future research. This information could potentially be collected in surveys and made publicly available in the future to facilitate studies that have the aim of gaining insight into how to select interviewers to increase HIV-testing participation rates [24].

Second, in our model we included an extensive set of potential predictors for HIV status and testing participation. However, there is a trade-off between guarding against bias by including additional covariates on the one hand, and the risk of overfitting and inefficiency on the other. Therefore, we recommend that researchers implementing this approach conduct sensitivity analyses to determine the extent to which point estimates and confidence intervals are sensitive to model specification. Here, we have examined whether our results are affected by adopting a more parsimonious model. We re-estimated the selection model and included only the following covariates: age (as a continuous variable), location, type of location of residence, distance to nearest road, marital status and month of interview. We find very similar HIV-prevalence estimates for both men (HIV prevalence of 26%, 95% CI 16–35) and women (HIV prevalence of 34%, 95% CI 27–40); therefore, the results in this case appear to be quite robust with respect to how the model is specified. Nevertheless, this type of sensitivity analysis should form an integral part of future research using this approach.

Finally, our results depend on the assumption that interviewers are as good as randomly allocated once we condition on observed characteristics of surveillance participants. Ultimately, this assumption cannot be tested with complete certainty because such a test would require counterfactual data (the HIV status of those who decline to test). In the case of this study it is, however, highly plausible that the assumption holds because the Africa Centre HIV surveillance allocates interviewers on the basis of the design of the survey such that interviewers are arbitrarily assigned to geographic sub-areas and not to potential individual participants. More generally, future studies could lend further empirical strength to the assumption that interviewers are as good as randomly assigned in particular surveillance settings, for example by validating interviewer identity against a randomly assigned variable that changes HIV-testing participation rates. We are working on such a validation study in Tanzania. Alternatively, collecting data on additional potential selection variables, such as detailed information on interviewer characteristics, would facilitate use of the selection model methodology. By providing researchers with the ability to generate a series of estimates derived from models with different selection variables, this would strengthen our capacity to assess the plausibility of the assumptions underlying the selection process. The SemiParBIVProbit R package used for the models adopted in this paper is publicly available, and designed to be easily implemented in a variety of settings affected by missing data [49]. Therefore, in conjunction with this software, these additional selection variables could be used to extend the application of selection models.

## Conclusions

Selection bias is a major concern in HIV surveys, particularly where rates of participation are low. Accounting for the fact that the relationship between HIV status and participation in testing is unknown, we find enlarged confidence intervals, which indicate that the point estimates for HIV prevalence in these situations are much more uncertain than previously thought. Our results illustrate the importance of correctly estimating this uncertainty and emphasize that it is critical to establish approaches that are effective at raising participation rates in HIV surveys that suffer from high levels of missingness.

Overall, this paper demonstrates the feasibility of implementing selection models in the context of health and demographic surveillance sites, and the approach we use here illustrates how to account for missing data when the assumption of MAR is unrealistic. As interviewer identity is routinely collected as paradata in epidemiological surveys, this approach has many practical applications, including, but not limited to, the estimation of HIV prevalence.
